# Exposure to ochratoxin a induces organelle damage during porcine oocyte maturation

**DOI:** 10.3389/fvets.2026.1912554

**Published:** 2026-08-06

**Authors:** Lin-Lin Hu, Yang Yi, Xing-He Ke, Min-Min Ou, Lan-Lan Nong, Li-Zhou Qin, Shao-Chen Sun, Caizhu Wang, Bi-Yun Liao

**Affiliations:** 1 Guangxi Key Laboratory of Artificial Intelligence for Genetic Diseases of Long-dwelling Nationalities, Affiliated Hospital of Youjiang Medical University for Nationalities, Baise, Guangxi, China; 2 College of Animal Science and Technology, Nanjing Agricultural University, Nanjing, China; 3 Center of Reproductive Medicine, Maternity and Child Health Care of Guangxi Zhuang Autonomous Region, Nanning, Guangxi, China

**Keywords:** cell cycle, meiosis, microtubule, ochratoxin A, oocyte

## Abstract

**Introduction:**

Ochratoxin A is a toxic mycotoxin produced by fungi, which is commonly detected in contaminated cereals and animal feeds. It exhibits nephrotoxicity, hepatotoxicity, genotoxicity, immunotoxicity, and reproductive toxicity.

**Methods:**

In the present study, we investigated the adverse effects of ochratoxin A on organelle distribution using a porcine oocyte model.

**Results:**

We demonstrated that in vitro exposure to ochratoxin A inhibited cumulus cell expansion and polar body extrusion in oocytes. Ochratoxin A reduced cortical endoplasmic reticulum distribution and increased GRP78 expression, indicating the induction of endoplasmic reticulum stress. In contrast, the Golgi apparatus exhibited abnormal accumulation, accompanied by decreased Rab7 expression and aberrant localization, suggesting impaired vesicular transport. Similarly, lysosomes accumulated in the cytoplasm of porcine oocytes following ochratoxin A treatment. We also found that mitochondrial function was disrupted, as evidenced by reduced ATP production and aberrant mitochondrial membrane potential.

**Conclusion:**

Taken together, these results indicate that ochratoxin A impairs organelle distribution, induces endoplasmic reticulum stress, and causes vesicular transport defects during porcine oocyte maturation.

## Introduction

Ochratoxin A (OTA) is a mycotoxin produced by fungi of the genera Aspergillus and Penicillium, which is commonly detected in cereals and feed. OTA is widely distributed in food commodities, crops, and related feed products worldwide (1). Common cereals, including corn, wheat, rice, sorghum, oats, and rye, are all susceptible to OTA contamination (2). OTA exerts multiple toxic effects on humans and mammals, mainly including nephrotoxicity, hepatotoxicity, immunotoxicity, reproductive toxicity, and neurotoxicity (3). OTA negatively impacts the fertility of livestock. Studies have shown that OTA exposure induces endocrine disorders in animals, such as elevated estradiol secretion and inhibited testosterone and cortisol secretion, thereby leading to reproductive dysfunction (4). OTA exposure also reduces sperm motility and count, impairing the reproductive performance of animals (5). In addition, OTA exposure compromises oocyte maturation, resulting in decreased polar body extrusion, and this effect is more pronounced in porcine oocytes than in mouse oocytes (6). Continuous OTA exposure reduces ovarian weight and the number of corpora lutea in pregnant rats and causes delayed puberty and ovarian development in F1 female offspring (7). In porcine oocytes, OTA exposure also induces oxidative stress, leading to aberrant cytoskeleton dynamics, autophagy, and apoptosis (8). The effects of OTA on the cell cycle arrest in mammalian oocytes were also reported (9), which other mycotoxins such as beauvericin and apicidin (10, 11). Therefore, OTA exposure impairs livestock fertility by compromising oocyte quality.

Organelles play crucial roles in oocyte maturation and function (12). Mitochondria supply essential ATP for oocyte meiosis, cytoplasmic maturation, and fertilization, and their distribution and activity directly determine oocyte quality. The endoplasmic reticulum (ER) regulates calcium homeostasis and protein folding, and ER stress can trigger oocyte apoptosis. ER stress is a cellular stress response induced by the accumulation of unfolded or misfolded proteins, which disrupts ER homeostasis. This stress activates the unfolded protein response (UPR) to restore ER function by promoting protein folding, degrading misfolded proteins, and reducing protein synthesis (13). ER stress and UPR dysfunction can lead to cellular apoptosis. In oocytes, ER stress is closely associated with impaired maturation, as it disturbs calcium homeostasis and protein processing, thereby compromising oocyte quality and reproductive competence (14). In the mycotoxin zearalenone-exposed porcine oocytes, ER stress occurred and GRP78 expression showed aberrant pattern (15); Besides, the emerging mycotoxin ustiloxin A also triggered ER stress and increased GRP78 expression in oocytes (16). The Golgi apparatus participates in protein synthesis, secretion, and lipid metabolism, supporting oocyte development. Rab family proteins are small GTPases that play pivotal roles in vesicular transport of proteins, lipids, and other molecules between organelles and the plasma membrane. Rab proteins localize to specific organelles or vesicular compartments and regulate vesicle formation, docking, and fusion by cycling between GTP-bound and GDP-bound states (17). Rab-mediated vesicular transport is essential for oocyte maturation due to its roles in ER–Golgi communication and intracellular homeostasis (18). Meanwhile, lysosomes maintain cellular homeostasis by degrading damaged organelles and macromolecules through autophagy, which is critical for oocyte survival and maturation. Therefore, these organelles function coordinately to ensure normal oocyte development and reproductive competence.

Although numerous studies have documented the other mycotoxins such as ZEN, AFB1, DON on the organelles function, and the multiple toxicities of OTA on oxidative stress using various models, particularly its disruptive effects on cytoskeleton dynamics in oocytes, its impacts on organelle-dependent cytoplasmic maturation remain largely unclear. In the present study, we used porcine oocytes as a model, given that their nuclear and cytoplasmic maturation is asynchronous. We demonstrated that OTA induces ER stress, causes vesicular transport defects, and impairs organelle distribution, providing novel insights into the reproductive toxicity of OTA in oocytes.

## Materials and methods

### Porcine oocyte collection and culture

Porcine ovaries from were collected from healthy sows (Duroc-Landrace-Yorkshire) (6–8 months old) immediately after slaughter at a local abattoir and transported to the laboratory within 2 h in physiological saline (37 °C) supplemented with 100 U/mL penicillin and 100 μg/mL streptomycin to prevent bacterial contamination. On each experimental day, we received 30–60 ovaries and collected approximately 20–40 oocytes from the mature follicles of each ovary. These oocytes were mixed and then randomly divided into different groups to avoid individual differences. All experiments were performed using the same procedure and replicated more than 3 times. All samples used in the tests were collected on the same day to ensure consistency. Upon arrival at the laboratory, the ovaries were washed three times with sterile phosphate-buffered saline (PBS, pH 7.4). Cumulus-oocyte complexes (COCs) were aspirated from 3 to 6 mm follicles using a 10 mL syringe fitted with a 21-gauge needle. The aspirated follicular fluid containing COCs was transferred to a 50 mL centrifuge tube and allowed to settle for 5 min at room temperature. COCs with uniform cytoplasm, an intact zona pellucida, and surrounded by at least three layers of cumulus cells were selected under a stereomicroscope. The selected COCs were washed three times in pre-warmed (37 °C) TCM-199 medium (containing amino acids, vitamins, inorganic salts, and other components; Thermo Fisher, Cat. No. 1150059) supplemented with 10% fetal bovine serum (FBS), 100 U/mL penicillin, and 100 μg/mL streptomycin, and then transferred to a 4-well culture plate containing the same medium for subsequent experiments. 44–48 h culture time for polar body extrusion; 24–28 h culture for organelle examination.

### Antibodies and chemicals

Ochratoxin A was purchased from Aladdin (O102402, Shanghai); Mito-tracker was from Maibo Bio (C1048); TMRE kit, Golgi-tracker green, Lyso-tracker Red were from Beyotime (C2001S-1; C1043, C1046); ATP kit was from Sigma-Aldrich (St. Louis, MO, United States); ER-Tracker blue was from Wobo Nanjing (LX3610); Rab7 Rabbit antibody (1:100), GRP78 rabbit antibody (1:50), GAPDH rabbit antibody (1:500), *α*-tubulin rabbit antibody (1:500) were from Protein Tech (11587–1, 55469–1, 10494–1, 11224–1, Wuhan); Rab10 rabbit antibody (1:100) was from Abcam (AB237703, Cambridge, United Kingdom). All other regents were purchased from Signal Aldrich.

### Ochratoxin a treatment

After selection, COCs were randomly divided into control and OTA-treated groups. OTA stock solution (50 mM) was prepared in dimethyl sulfoxide (DMSO) and diluted in maturation medium to the desired working concentration (5, 7.5, and 10 μM) were set with the final DMSO concentration maintained below 0.1% in all groups to avoid solvent-induced effects. The dose selection was due to our previous study (8). COCs in the treated group were cultured *in vitro* in TCM-199 maturation medium supplemented with OTA, while control COCs were cultured in identical medium without OTA but with the same dose of DMSO. All oocytes were cultured at 38.5 °C under a humidified atmosphere of 5% CO₂ in air for a continuous 44–48 h to allow *in vitro* maturation. Following maturation culture, oocytes were collected for subsequent morphological evaluation and molecular analysis. The maturation status of porcine oocytes was determined based on the expansion degree of cumulus cells and the extrusion rate of the first polar body. We define the expansion degree as the ratio of the diameter of the expanded granulosa cell mass to the diameter of the oocyte.

### Immunofluorescence staining

GRP78 is one of the major ER chaperones in cells and serves as a reliable indicator of ER stress levels. Rab10 and Rab7 are key proteins involved in vesicular transport. Immunofluorescence procedures were performed according to our previously published protocol (19). Following *in vitro* maturation and OTA treatment, cumulus cells were removed from porcine oocytes by gentle pipetting, and oocytes were fixed in 4% paraformaldehyde in PBS for 30 min at room temperature. Fixed oocytes were permeabilized with 1% Triton X-100 in PBS for 20 min and blocked in 3% bovine serum albumin (BSA) for 1 h to minimize nonspecific binding. Oocytes were then incubated with primary antibodies (diluted according to the manufacturer’s instructions) overnight at 4 °C. After three washes in PBS containing 0.1% Tween-20 (PBST), oocytes were incubated with TRITC-conjugated secondary antibodies for 1 h at room temperature in the dark. Nuclei were stained with 5 μg/mL Hoechst 33342 in PBS for 5 min. Finally, oocytes were mounted on glass slides and examined under a laser scanning confocal microscope. Images were acquired and analyzed using ZEN (Zeiss) and ImageJ (NIH) software.

### Western blot

For western blot analysis, a total of 150–200 porcine oocytes per group were collected and lysed in RIPA lysis buffer supplemented with protease and phosphatase inhibitors to prevent protein degradation. Protein concentration was determined using a BCA protein assay kit. Equal amounts of protein samples were separated by sodium dodecyl sulfate-polyacrylamide gel electrophoresis (SDS-PAGE) and transferred onto polyvinylidene fluoride (PVDF) membranes. The membranes were blocked with 5% non-fat milk in Tris-buffered saline containing Tween-20 (TBST) for 1 h at room temperature, then incubated with primary antibodies overnight at 4 °C. After three washes with TBST, the membranes were incubated with horseradish peroxidase (HRP)-conjugated secondary antibodies for 1 h at room temperature. Protein bands were visualized using an enhanced chemiluminescence (ECL) detection system.

### Fluorescence intensity analysis by ImageJ

Fluorescence images of oocytes were captured using a laser scanning confocal microscope and analyzed for fluorescence intensity with ImageJ software (version 1.8.0). All images were processed using identical parameters (brightness and contrast) to ensure consistency. For each oocyte, a region of interest (ROI) was manually drawn to cover the entire oocyte cytoplasm or the specific target region, avoiding non-specific fluorescence and background noise. The mean gray value of the ROI was measured, and background fluorescence intensity, obtained from an acellular region in the same image, was subtracted to yield the actual fluorescence intensity of the target. At least 15 oocytes, with a maximum of 60 oocytes, were analyzed per group. Relative fluorescence intensity was calculated and normalized to the control group for subsequent statistical analysis.

### Statistical analysis

All experimental data were presented as mean ± standard error of the mean (SEM) from at least three independent replicates. The independence indicates that all the samples or treatment was different with other repeat. Statistical analysis was performed using GraphPad Prism software (version 8.0). Differences between multiple groups were compared by one-way analysis of variance (ANOVA) followed by Tukey’s *post-hoc* test, while differences between two groups were analyzed by unpaired two-tailed Student’s *t*-test. A *p* < 0.05 was considered statistically significant.

## Results

### OTA inhibits cumulus cell expansion and polar body extrusion of porcine oocytes

**Figure 1:**
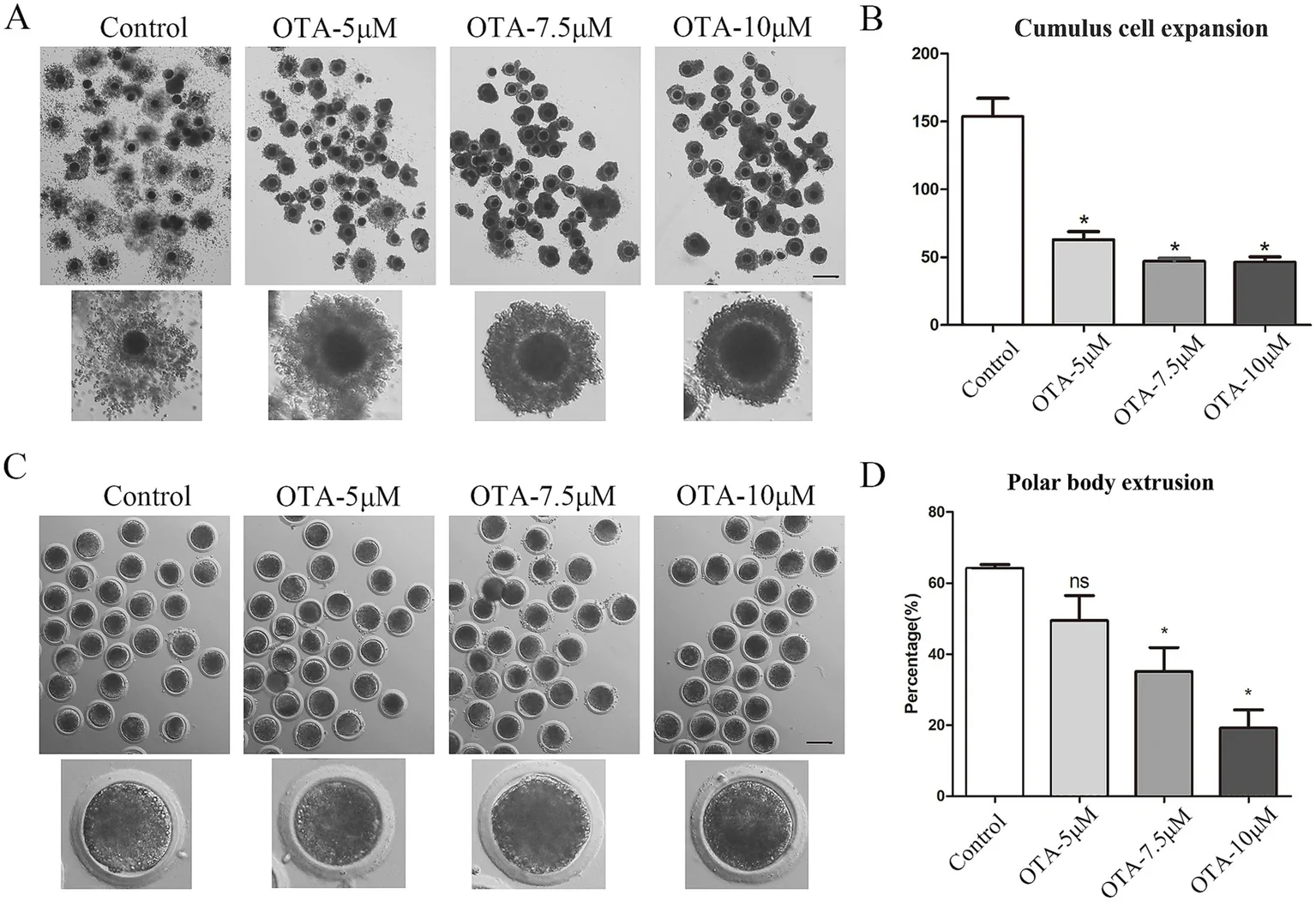
OTA inhibits cumulus cell expansion and oocyte maturation of pigs. **(A)** Porcine COCs were cultured 44–48 h. Cumulus granulosa cell diffusion was observed in the control and treatment groups. Bar = 200 μm. **(B)** Diffusion degree of cumulus granulosa cells in the control and treatment groups. *p < 0.05. **(C)** Typical figure of the first polar body extrusion in the control and treatment groups. Bar = 100 μm. **(D)** First polar body extrusion rate in the control and treatment groups. Ns, *p* > 0.05; *p < 0.05.

First, cumulus-oocyte complexes (COCs) were divided into a control group and three OTA-treated groups (30–60 COCs per group). After 44–48 h of *in vitro* culture, the expansion degree of cumulus cells in both the control and OTA-treated groups was observed under a microscope, and the results indicated that cumulus expansion was disrupted (Figure 1A). We found that the cumulus expansion degree in the OTA-treated groups was significantly lower than that in the control group, and it decreased with increasing OTA concentration (control group: 153.50 ± 13.58 μm, *n* = 66; 5 μM OTA-treated group: 62.80 ± 6.00 μm, *n* = 76, *p* < 0.05; 7.5 μM OTA-treated group: 46.95 ± 2.32 μm, *n* = 68, *p* < 0.05; 10 μM OTA-treated group: 46.18 ± 4.09 μm, *n* = 74, *p* < 0.05) (Figure 1B). Subsequently, the extrusion of the first polar body in oocytes with cumulus cells removed was observed (Figure 1C). We found that the extrusion rate of the first polar body in the control group was significantly higher than that in the OTA-treated groups (control group: 64.13 ± 1.07 %, *n* = 142; 5 μM OTA-treated group: 49.43 ± 7.03 %, *n* = 146, ns; 7.5 μM OTA-treated group: 35.07 ± 6.78 %, *n* = 150, *p* < 0.05; 10 μM OTA-treated group: 19.17 ± 5.20 %, *n* = 136, *p* < 0.05) (Figure 1D). Due to the effects of OTA on cumulus cell and oocyte polar body extrusion, we chose 10 μM for the following experiments since this dose both effectively disturbed cumulus expansion and polar body extrusion.

### OTA alters ER accumulation and GRP78 expression in porcine oocytes

**Figure 2:**
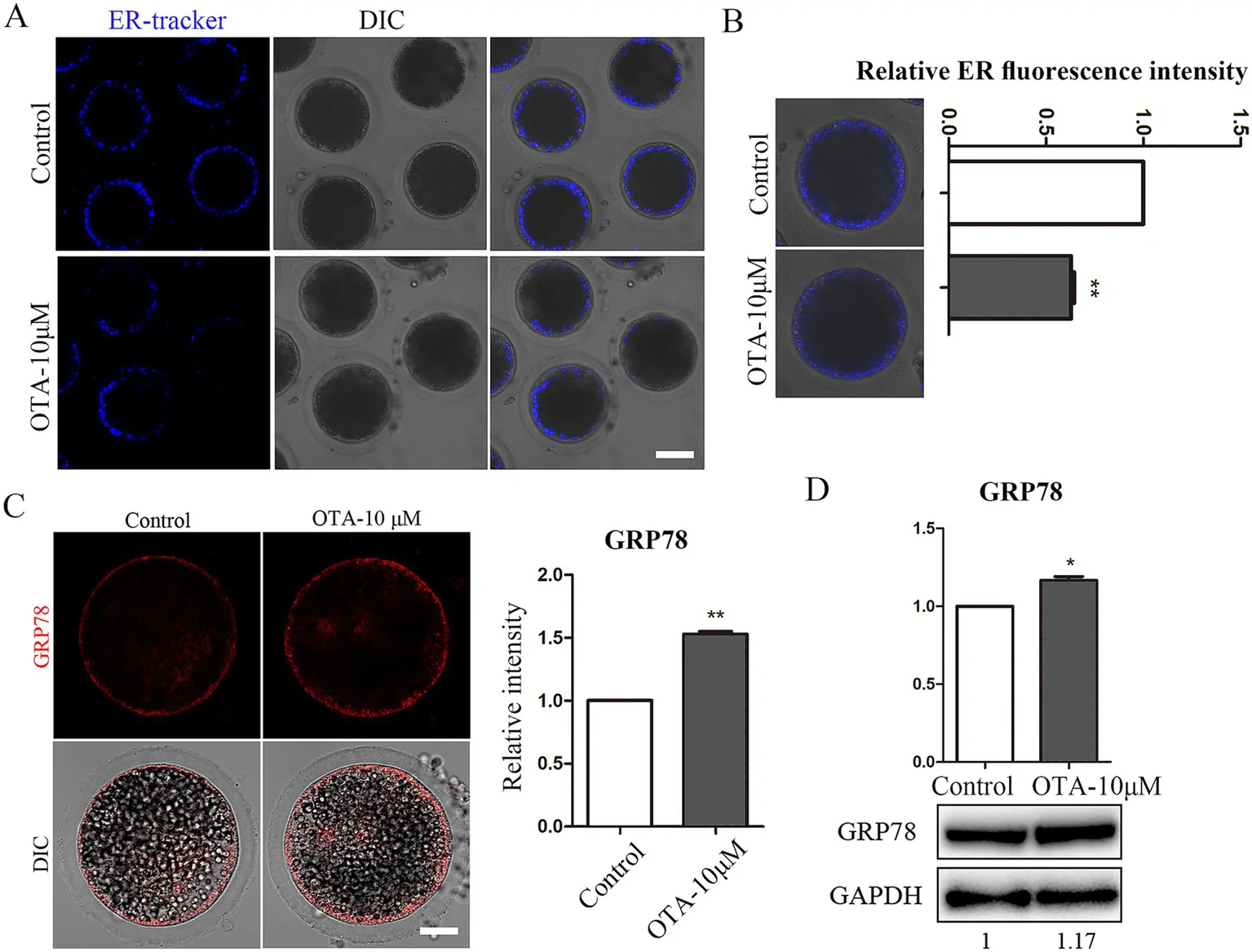
OTA induces GRP78-based ER stress in porcine oocytes. **(A)** Endoplasmic reticulum fluorescence staining showed that the fluorescence of treatment group was significantly decreased. Blue, endoplasmic reticulum. Bar = 50 μm. **(B)** The results of statistical analysis showed that the intensity of endoplasmic reticulum in treatment group was significantly reduced. **p < 0.01. **(C)** GRP78 fluorescence staining results showed that the fluorescence of treatment group was obviously higher. Red, GRP78. Bar = 20 μm. Statistical analysis results showed that the fluorescence of GRP78 in treatment group increased significantly. **p < 0.01. **(D)** Western Blot analysis of GRP78 protein expression in the control and treatment groups. *p < 0.05.

The endoplasmic reticulum (ER) plays a crucial role in maintaining protein homeostasis during porcine oocyte maturation. In this section, ER-Tracker was used to visualize ER distribution in oocytes. Fluorescence imaging (Figure 2A) revealed that ER fluorescence intensity was significantly lower in the OTA-treated group than in the control group, which was further supported by quantitative analysis (control intensity set to 1, *n* = 52; OTA-treated group: 0.63 ± 0.02, *n* = 58, *p* < 0.01) (Figure 2B). To assess the effect of OTA exposure on ER stress, we examined the expression and localization of GRP78. Fluorescence microscopy showed markedly higher GRP78 intensity in the OTA-treated group compared with the control group. Statistical quantification (Figure 2C) confirmed this observation (control set to 1, *n* = 51; OTA-treated group: 1.53 ± 0.02, *n* = 53, *p* < 0.01). We further detected GRP78 protein expression by Western blotting. As shown in Figure 2D, GRP78 protein levels were significantly elevated in the OTA-treated group relative to the control group (control set to 1; OTA-treated group: 1.17 ± 0.02, *p* < 0.05).

### OTA disturbs Golgi apparatus and Rabs accumulation in porcine oocytes

**Figure 3:**
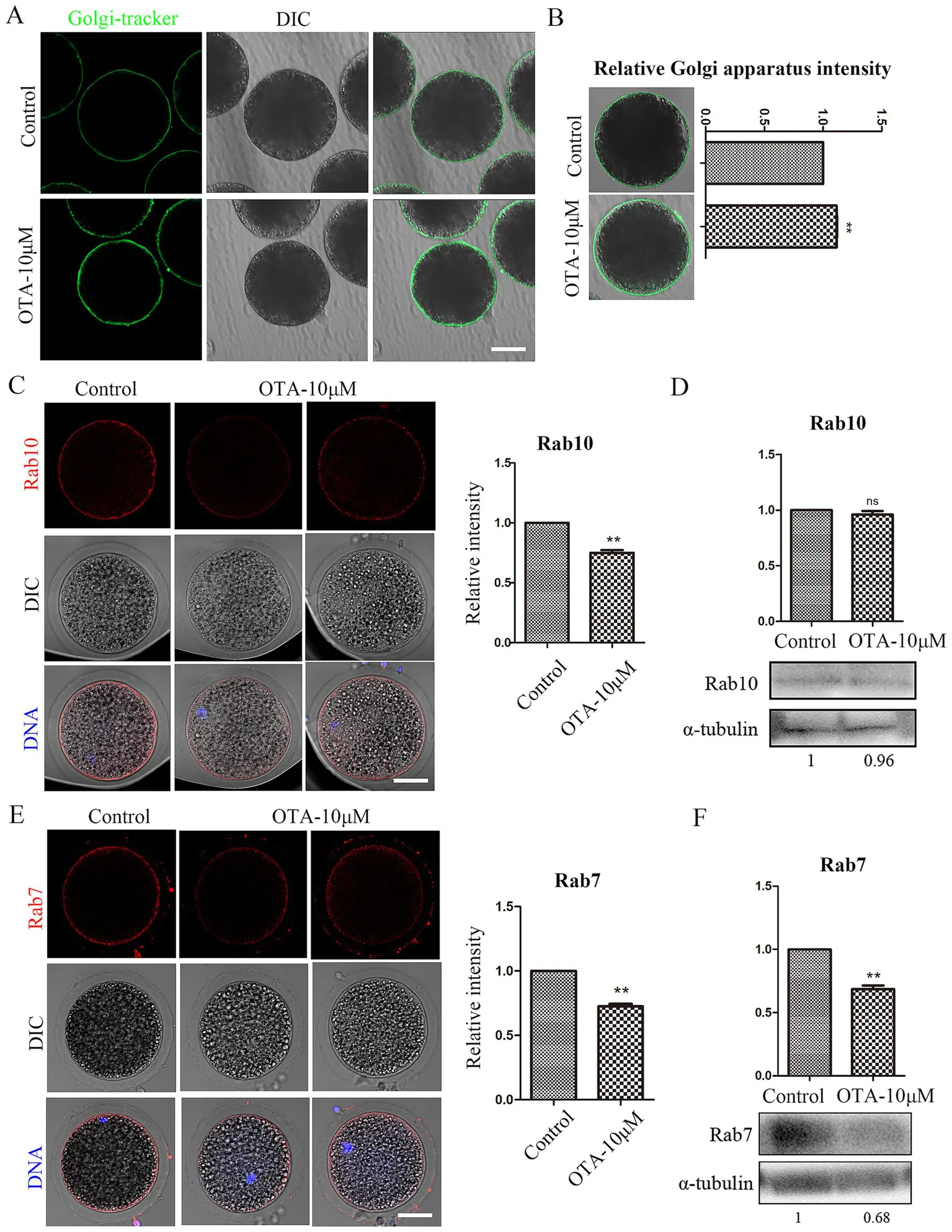
OTA disturbs vesicular transport from Golgi apparatus in porcine oocytes. **(A)** The Golgi fluorescence staining showed that the fluorescence of treatment group was significantly higher than the control group. Green, Golgi apparatus. Bar = 40 μm. **(B)** Statistical analysis showed that the fluorescence intensity of Golgi apparatus in treatment group was significantly higher than that in the control group. **p < 0.01. **(C)** The fluorescence staining results of Rab10 showed that its fluorescence in the treatment group was obviously weaker than that of treatment group. Red, Rab10. Bar = 30 μm. The results of statistical analysis showed that the fluorescence intensity of Rab10 in treatment group decreased significantly. **p < 0.01. **(D)** Western blot analysis of Rab10 in the control and treatment group showed no significant changes in Rab10 protein expression. Ns, *p* > 0.05. **(E)** The Rab7 fluorescence staining results showed that the fluorescence in the treatment group was significantly weaker. Red, Rab7. Bar = 30 μm. **(F)** Western blot analysis of Rab7 protein expression in the control and treatment group. **p < 0.01.

The Golgi apparatus plays a central role in intracellular vesicle trafficking and is essential for porcine oocyte maturation. Golgi-Tracker was employed to assess Golgi apparatus distribution in porcine oocytes. As shown in Figure 3A, Golgi fluorescence intensity was significantly higher in the OTA-treated group than in the control group. Statistical analysis (Figure 3B) confirmed this observation (control set to 1, *n* = 58; OTA-treated group: 1.12 ± 0.01, *n* = 50, *p* < 0.01). To determine whether OTA impairs Golgi function, we evaluated the effects of OTA exposure on Rab7 and Rab10. Representative Rab10 fluorescence images (Figure 3C) showed that Rab10 intensity was markedly lower in the OTA-treated group than in the control group. Statistical quantification verified this finding (control set to 1, *n* = 45; OTA-treated group: 0.75 ± 0.02, *n* = 45, *p* < 0.01). However, Western blot analysis (Figure 3D) revealed no significant change in Rab10 protein expression. We next examined the effect of OTA exposure on Rab7. Fluorescence imaging (Figure 3E) demonstrated that Rab7 intensity was significantly lower in the OTA-treated group compared with the control group. Quantitative analysis (Figure 3E) supported this result (control set to 1, *n* = 50; OTA-treated group: 0.73 ± 0.02, *n* = 50, *p* < 0.02). Western blot analysis of Rab7 (Figure 3F) showed that its protein level was significantly decreased following OTA treatment (control set to 1; OTA-treated group: 0.68 ± 0.03, *p* < 0.01). These results indicate that OTA exposure affects the fluorescent localization of both Rab7 and Rab10, but only alters the protein expression of Rab7, not Rab10. Therefore, we speculate that OTA-induced Golgi apparatus dysfunction is more closely associated with Rab7.

### OTA induces aberrant accumulation of lysosomes in porcine oocytes

**Figure 4:**
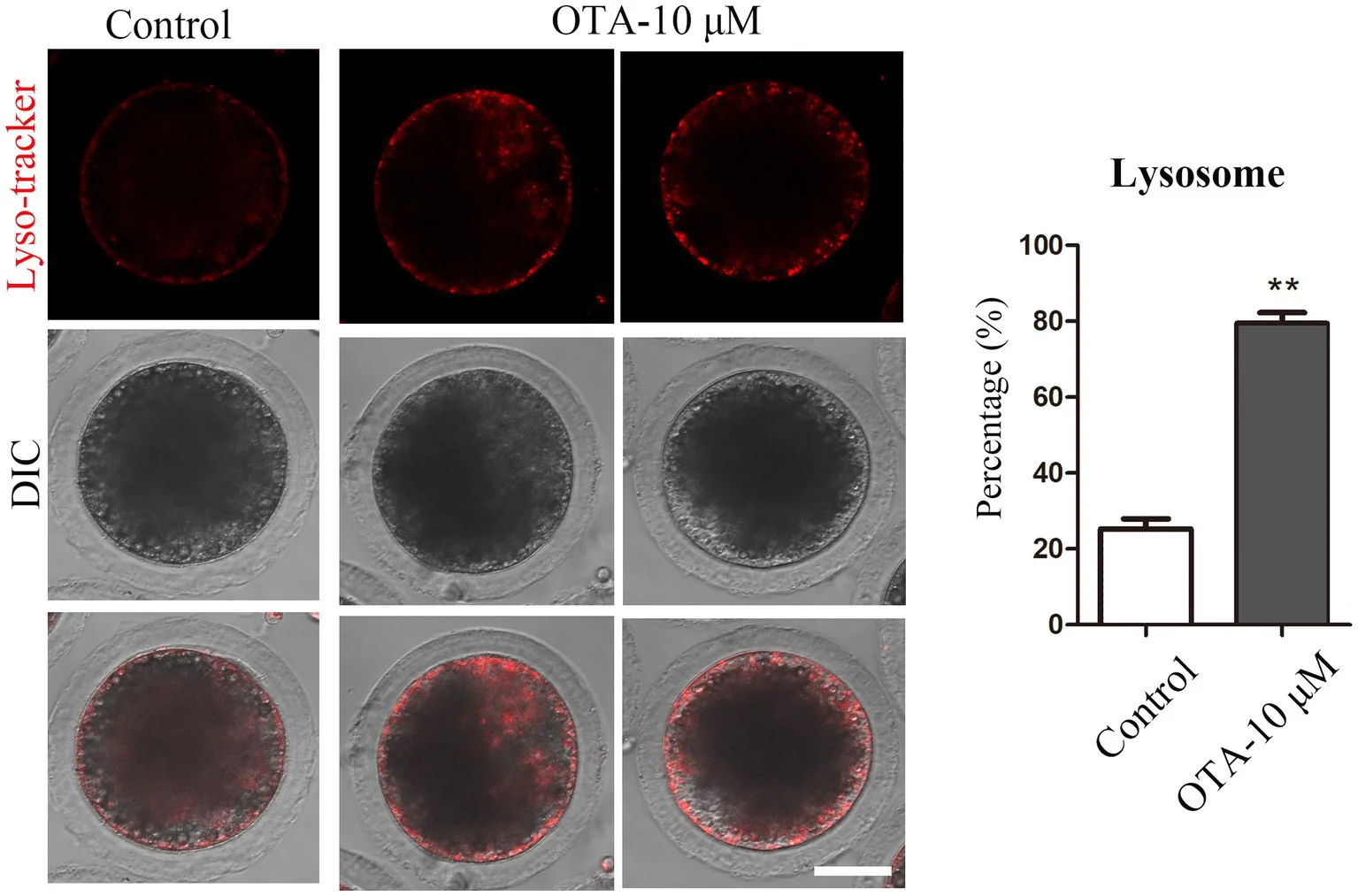
OTA induces aberrant accumulation of lysosomes in porcine oocytes. The fluorescence staining results of lysosome showed that the lysosome localization of treatment group was abnormal. Red, lysosome. Bar = 30 μm. Statistical analysis results showed that the abnormal localization rate of lysosomes in the treatment group increased significantly. **p < 0.01.

The lysosome serves as the metabolic center of the cell and plays a crucial role in maintaining intracellular protein homeostasis. In this section, LysoTracker was used to detect lysosomes in porcine oocytes. As shown in the fluorescence images (Figure 4), abnormal lysosomal accumulation was observed in the OTA-treated group. Subsequently, we quantified the rate of abnormal lysosomal accumulation in the control and OTA-treated groups. As presented in Figure 4 (we set the average fluorescence intensity of control group as 1, while 1.5 was considered as abnormal accumulation), the percentage of oocytes with abnormal lysosomal accumulation was significantly higher in the OTA-treated group than in the control group (control: 25.28 ± 2.67%, *n* = 63; OTA-treated: 79.49 ± 2.77%, *n* = 63, *p* < 0.01). These results indicate that OTA exposure disrupts lysosomal distribution in porcine oocytes.

### OTA disrupts mitochondria distribution and MMP in porcine oocytes

**Figure 5:**
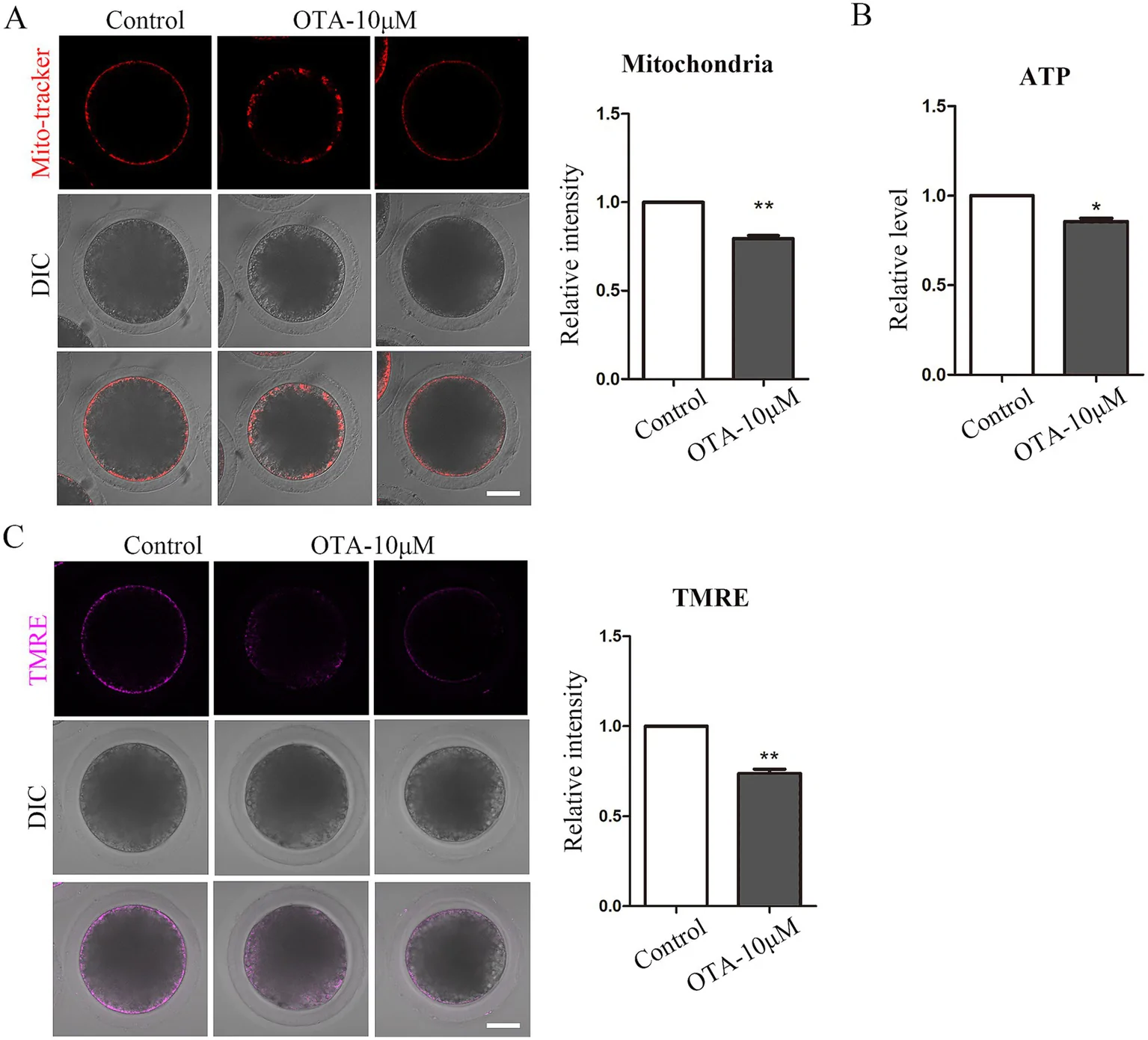
OTA causes mitochondria dysfunction in porcine oocytes. **(A)** Mitochondrial fluorescence signals of control group were significantly higher than that of treatment group. Red, mitochondria. Bar = 30 μm. Statistical analysis results showed that the fluorescence intensity of control group was significantly higher than that of treatment group. **p < 0.01. **(B)** The ATP level of control group was higher than that of treatment group. *p < 0.05. **(C)** TMRE fluorescence staining results showed that the fluorescence in the treatment group was lower than of control group. Pink, TMRE. Bar = 30 μm. The fluorescence analysis results of TMRE showed that the intensity of treatment group decreased significantly. **p < 0.01.

Mitochondria, as the primary energy-supplying organelles, play a vital role during oocyte maturation. In this section, MitoTracker was used to evaluate the effect of OTA exposure on mitochondrial distribution in porcine oocytes. Live mitochondrial staining revealed that mitochondrial fluorescence intensity was markedly lower in the OTA-treated group than in the control group (Figure 5A). Statistical analysis confirmed this observation (control intensity set to 1, *n* = 35; OTA-treated group: 0.79 ± 0.02, *n* = 37, *p* < 0.01) (Figure 5A). ATP level serves as a key indicator of mitochondrial function. ATP concentrations in oocytes were measured using an ATP bioluminescence assay kit. Statistical analysis showed that ATP levels were significantly lower in the OTA-treated group compared with the control group (control set to 1; OTA-treated group: 0.86 ± 0.02, *p* < 0.05) (Figure 5B). Mitochondrial membrane potential (MMP) was assessed using TMRE staining. Fluorescence imaging revealed markedly reduced TMRE intensity in the OTA-treated group (Figure 5C), which was further validated by quantitative analysis (control intensity set to 1, *n* = 34; OTA-treated group: 0.74 ± 0.02, *n* = 34, *p* < 0.01) (Figure 5C).

## Discussion

In this study, we used an *in vitro* model to investigate the effects of OTA exposure on the distribution of multiple organelles during porcine oocyte maturation. Given individual variations among pigs, we performed in vitro experiments rather than *in vivo* treatments to directly reflect the intrinsic effects of OTA on oocytes. Although further in vivo evidence is still required, our current results demonstrate that OTA exposure directly impairs porcine oocyte maturation and exerts toxic effects on mitochondria, the endoplasmic reticulum, Golgi apparatus, and lysosomes, thereby reducing oocyte quality and compromising reproductive competence.

We first demonstrated that OTA concentrations ranging from 5 to 10 μM all disrupted cumulus expansion, while 7.5 and 10 μM OTA significantly inhibited porcine oocyte maturation. These doses fall into the scope of dose under the environmental exposure in the contaminated feeds; while in the blood samples, 1–10 μM were detected for acute toxicity experiments in pigs. In addition, OTA exposure has also been shown to impair early embryonic development in a porcine model (20). In mice, in vivo OTA exposure targets the ovary and disrupts follicular development (21); and in vitro studies have confirmed that OTA impairs the meiotic progression of mouse oocytes (22). Previous studies have reported that OTA induces oxidative stress, apoptosis, and autophagy in porcine oocytes (8). While spindle disruption generally induces the aberrant distribution of organelles since at the metaphase I of oocytes organelles are around the spindle; and organelles localization is dynamics depended on difference cell cycle stage. However, its effects on organelle function remain incompletely understood. We therefore explored the impacts of OTA exposure on multiple organelles in the present study.

Although there were several previous studies on the potential mechanism of mycotoxins on the oocytes, the current data mostly by RNA-seq indicated that their toxic effects are very general and can affect multiple systems, which is hard to clarify the specific molecular pathways. We then mainly focused its effects on organelles. The endoplasmic reticulum (ER) is essential for oocyte maturation, since protein synthesis, transport, and folding during oocyte maturation depend on ER function (23). In the present study, we observed aberrant ER distribution and the induction of ER stress in porcine oocytes following OTA exposure. These findings are consistent with previous reports showing that OTA upregulates GRP78 expression and triggers ER stress in mouse and rat glomerular mesangial cells, as well as in porcine kidney and spleen tissues (24, 25). Another study demonstrated that OTA exposure increases the expression of ER stress markers including GRP78 in human gastric epithelial cells (26), further supporting our results. Similarly, other mycotoxins can also induce ER stress in oocytes; for instance, HT-2 toxin impairs porcine oocyte quality by altering GRP78 expression (27). The Golgi apparatus mediates the transport and maturation of proteins and lipids along the secretory pathway from the ER. In oocytes, the Golgi apparatus is critical for maintaining protein homeostasis and is functionally linked to mitochondrial activity, and Golgi stress can trigger mitochondria-mediated apoptosis (28). We found that OTA altered Golgi distribution and impaired vesicular transport function, as reflected by aberrant localization of both Rab7 and Rab10 in porcine oocytes. Rab7 and Rab10 belong to the Rab GTPase superfamily and participate in Golgi-mediated vesicular trafficking. Rab10 regulates cortical granule transport during mouse oocyte fertilization (29), whereas Rab7 modulates actin organization, mitochondrial function, and mitophagy in oocytes (30, 31). Mycotoxins including OTA commonly disrupt Golgi function in mammalian oocytes (9). For example, deoxynivalenol exposure induces Golgi reorientation and alters GM130 expression (32). In addition, nivalenol (NIV) causes abnormal Golgi accumulation in mouse oocytes accompanied by upregulated Rab11 expression (33). Therefore, OTA and other mycotoxins may similarly impair ER and Golgi apparatus function in mammalian oocytes.

Lysosomes are dynamic, heterogeneous organelles that function as the degradation, metabolic, and signaling centers of the cell. In this study, we found that OTA exposure disrupted lysosomal distribution. These findings are consistent with previous reports showing that citrinin exposure induces abnormal lysosomal accumulation in mouse oocytes, accompanied by upregulated expression of the lysosomal marker LAMP2 (34). Lysosomal function in oocytes is closely associated with apoptosis, and mitochondrial dysfunction can trigger oxidative stress, which further promotes apoptotic progression. This mechanism is conserved in oocytes, ovaries, and granulosa cells (35, 36). Consistent with this, our results demonstrated that OTA exposure induced aberrant mitochondrial distribution, decreased mitochondrial membrane potential, and reduced ATP content, indicating that OTA impairs mitochondrial and lysosomal function and may induce apoptosis. These observations align with previous studies conducted in mouse, porcine, and ovine models (37, 38). The toxic effects of OTA on mitochondria have been documented across multiple systems. For instance, in human astrocytes, OTA exerts neurotoxicity by targeting mitochondria to induce apoptosis and calcium overload (39), with a proposed mechanism involving OTA-mediated promotion of glycolysis, mitochondrial dysfunction, and ferroptosis (40). In oocyte models, other mycotoxins elicit similar mitochondrial toxicity. Porcine oocytes exposed to nivalenol exhibit mitochondrial dysfunction characterized by aberrant ATP production and altered mitochondrial membrane potential (41). *In vivo* dietary exposure to multiple mycotoxins in mice also causes abnormal mitochondrial distribution in oocytes (42). In sheep, the mycotoxin beauvericin disrupts mitochondrial function during early embryonic development (43).

Taken together, these data collectively indicated that ochratoxin A could disrupt organelle distribution, induces ER stress and vesicular transport defects for porcine oocyte maturation.

## Data Availability

The original contributions presented in the study are included in the article/supplementary material, further inquiries can be directed to the corresponding authors.
